# Deep sequencing reveals distinct microRNA-mRNA signatures that differentiate pancreatic neuroendocrine tumor from non-diseased pancreas tissue

**DOI:** 10.1186/s12885-025-14043-w

**Published:** 2025-04-11

**Authors:** N Matyasovska, N Valkova, M Gala, S Bendikova, A Abdulhamed, V. Palicka, Neil Renwick, Pavol Čekan, Evan Paul

**Affiliations:** 1 MultiplexDX, s.r.o, Comenius University Science Park, Bratislava, Slovakia; 2 MultiplexDX, Inc, Rockville, MD USA; 3 Institute of Clinical Biochemistry and Diagnostics, Faculty of Medicine in Hradec Kralove, University Hospital, Charles University, Hradec Kralove, Czech Republic; 4 Laboratory of Translational RNA Biology, Department of Pathology and Molecular Medicine, Queen’s University, Kingston, Canada; 5 Laboratory of RNA Molecular Biology, The Rockefeller University, New York, NY USA

**Keywords:** Pancreatic neuroendocrine tumors, MicroRNA, mRNA, Biomarkers, miR-mRNA interaction networks

## Abstract

**Background:**

Only a limited number of biomarkers guide personalized management of pancreatic neuroendocrine tumors (PanNETs). Transcriptome profiling of microRNA (miRs) and mRNA has shown value in segregating PanNETs and identifying patients more likely to respond to treatment. Because miRs are key regulators of mRNA expression, we sought to integrate expression data from both RNA species into miR-mRNA interaction networks to advance our understanding of PanNET biology.

**Methods:**

We used deep miR/mRNA sequencing on six low-grade/high-risk, well-differentiated PanNETs compared with seven non-diseased tissues to identify differentially expressed miRs/mRNAs. Then we crossed a list of differentially expressed mRNAs with a list of in silico predicted mRNA targets of the most and least abundant miRs to generate high probability miR-mRNA interaction networks.

**Results:**

Gene ontology and pathway analyses revealed several miR-mRNA pairs implicated in cellular processes and pathways suggesting perturbed neuroendocrine function (miR-7 and *Reg* family genes), cell adhesion (miR-216 family and *NLGN1*, *NCAM1*, and *CNTN1*; miR-670 and the claudins, *CLDN1* and *CLDN2*), and metabolic processes (miR-670 and *BCAT1*/*MPST*; miR-129 and *CTH*).

**Conclusion:**

These novel miR-mRNA interaction networks identified dysregulated pathways not observed when assessing mRNA alone and provide a foundation for further investigation of their utility as diagnostic and predictive biomarkers.

**Supplementary Information:**

The online version contains supplementary material available at 10.1186/s12885-025-14043-w.

## Introduction

Neoplasms arising from neuroendocrine cells within pancreatic islets are known as pancreatic neuroendocrine neoplasms (PanNENs), which can be divided into two broad histopathological categories that account for both the level of differentiation and proliferation: pancreatic neuroendocrine tumors (PanNETs) have a well-differentiated morphology and are low to high grade (G1-G3), whereas pancreatic neuroendocrine carcinomas (PanNECs) have a poorly differentiated morphology and are high grade (G3). PanNETs comprise over 90% of PanNENs and even though PanNETs are rare, comprising < 2% of all pancreatic cancers, their incidence has increased in recent decades, emphasizing the need to understand their molecular, clinical, and pathological characteristics [1, 2, 3].

Current methods such as counting mitotic cells and Ki67 labeling are essential to determine tumor grade, but these methods have limitations. The Ki67 index, used for classification, may not always show the aggressiveness of the PanNETs, raising concerns that patients with high Ki67 G1/G2 PanNETs might receive suboptimal treatment. Because immunohistochemistry (IHC) for hormones and other biomarkers remains optional in the evaluation of PanNETs, more reliable links between tumor biology and clinical symptoms are needed. Therefore, there is a growing demand for biomarkers that better correlate tumor characteristics with patient symptoms to effectively guide treatment decisions. Biomarkers such as chromogranin A, synaptophysin, somatostatin receptors, *DAXX*/*ATRX*, p53/pRb, and *MGMT* help differentiate PanNET subtypes and improve diagnostic accuracy [4, 5, 6, 7], although some biomarkers associated with specific mutations (e.g., loss of *DAXX/ATRX/MEN1/TSC2/*p53/Rb) are more relevant for high-grade PanNETs [8, 9, 10, 11]. Uncovering new markers by looking at miR-mRNA interaction networks holds promise to advance our understanding of PanNET biology, especially in cases of low grade, well differentiated PanNETs where conventional markers like Ki67 are insufficient [6, 11, 12].

MicroRNAs (miRs) are small RNA molecules that negatively regulate gene expression by binding to the 3’ untranslated region (3’UTR) of target messenger RNAs (mRNAs) and either initiating cellular processes for transcript degradation or repressing protein translation [13]. Dysregulation of specific miRs can lead to aberrant expression of genes/proteins involved in key cellular processes, such as cell proliferation, apoptosis, and differentiation [14, 15]. Despite limited data concerning miR expression in PanNETs, several studies have discovered specific miRs and their regulatory networks that are crucial to the development and progression of these tumors, and they may be useful as diagnostic, prognostic, and therapeutic biomarkers [16, 17, 18, 19, 20, 21, 22]. PanNETs can be classified into distinct subtypes based on their unique miR and mRNA transcriptomes, and miR-based subtypes show concordance and enrichment in select mRNA subtypes, which underlines the close regulatory relationships between miRs and mRNA [23]. This raises an intriguing possibility that combining information from both the small noncoding (miR) and protein coding (mRNA) transcriptomes may yield even deeper insights into molecular heterogeneity and pathways involved in PanNET pathogenesis.

In this study, we aimed to demonstrate the feasibility of utilizing both mRNA and miR sequencing to identify markers and pathways that are altered in PanNETs. By analyzing mRNA and miR profiles in six PanNETs and seven non-diseased pancreatic tissue samples using deep sequencing, we provide a proof-of-concept analysis that can serve as a foundation for future retrospective or prospective studies on larger cohorts. Such studies could potentially reveal new prognostic and predictive biomarkers for PanNETs, contributing to improved classification and personalized treatment strategies.

## Materials and methods

### Patient cohort

The PanNET tissue sections were provided by the Ontario Tumour Bank, which is supported by the Ontario Institute for Cancer Research through funding provided by the Government of Ontario. The clinical characteristics of the patients’ tumor samples (*n* = 6) and non-diseased tissues (*n* = 7) are described in Supplementary Table S1. Usage of the PanNET samples were approved for research purposes through the Queen’s University Health Sciences & Affiliated Teaching Hospitals Research Ethics Board (PATH-145-14). Considering that the tissue could harbor molecular alterations responsible for the neoplastic degeneration in the ductal compartment, a pathologist delineated and dissected the non-diseased pancreatic tissue from pancreatic adenocarcinoma tissues, and the non-diseased tissue was used for further analyses. Non-diseased pancreatic tissues were obtained from the Surgical Pathology Archive in the Department of Pathology and Molecular Medicine at Queen’s University. Samples were approved for research through Research Ethics Board at Queen’s University (PATH-145-14). Hematoxylin-eosin-stained tissue sections from each case were reviewed by an experienced pathologist (AA) to assess tissue architecture and confirm neuroendocrine morphology. Grading was performed separately on Ki-67-stained materials, following the European Neuroendocrine Tumor Society criteria adopted by the World Health Organization classification in 2010. Two to five 5 μm sections of the six FFPE PanNETs were scraped from slides and deparaffinized using Paraffin Dissolver. Additionally, seven FFPE blocks of non-diseased tumor tissues were sectioned (5 sections of 5 μm) for RNA extraction.

### Total RNA isolation

Although the formalin-fixation and paraffin-embedding process in FFPE tissues results in chemical modifications and degradation of RNA and thus could be perceived as a weakness, advances in commercial assays for RNA extraction and library preparation have been tailored for FFPE tissues and can help to reverse or mitigate some of the tissue alterations caused by the FFPE process. This ultimately allows users to generate sequencing libraries with low input, low quality FFPE tissues and high-quality microRNA and RNA expression data. Using FFPE tissues has additional advantages because they are routinely collected, can be stored for long durations at room temperature at low cost, and facilitate retrospective studies with more complete longitudinal clinical data. Total RNA including small RNAs was isolated from all PanNETs and non-diseased tissue samples in a single batch using the NucleoSpin totalRNA FFPE XS kit (Macherey Nagel) according to the manufacturer’s protocol with the following modifications as recommended in the user instructions: (I) extension of the proteinase K digestion step to 90 min at 56 °C, and (II) inclusion of the optional on-column DNase digestion step to eliminate residual DNA. RNA quantity was determined using the Qubit RNA HS Assay Kit (Qubit 4 Fluorometer) and RNA quality was assessed using the Agilent High Sensitivity RNA ScreenTape (Agilent 4150 TapeStation). Samples with DV200 > 25% were considered suitable for library preparation.

### RNA library preparation and sequencing

Total RNA libraries were prepared using TAKARA SMARTer Stranded total RNA-Seq kit v3– Pico Input Mammalian library prep according to the manufacturer’s guidelines, with specific modifications recommended for highly degraded FFPE specimens, including (I) total RNA input 10 ng, (II) DNase pretreatment step, (III) omission of the fragmentation step, (IV) performing 10 PCR cycles for the first PCR amplification step, and (V) conducting 10 PCR cycles for the second PCR amplification step for 10 ng input. Following library preparation, the quantity and range of fragment sizes were evaluated using the Qubit dsDNA HS kit with a Qubit 4 Fluorometer, as well as the Agilent High Sensitivity DNA ScreenTape kit with an Agilent 4150 TapeStation.

The individual sequencing libraries were indexed using TruSeq Unique dual (UD) index adapters. Pooled libraries were spiked with 10% PhiX, as recommended by both Illumina and Takara and sequenced by an Illumina NovaSeq 6000 platform on an SP patterned flow cell. Paired-end sequencing with a read length of 2 × 100 bp was performed, aiming for approximately 100 million reads per sample.

### Small RNA library preparation and sequencing

Small RNA cDNA libraries were generated using QIAseq miR library kit (Illumina, San Diego, CA, USA) with QIAseq miR NGS 48 Indexes, implementing 16 amplification cycles for 100 ng input, and 19 PCR cycles for 10 ng input. All steps from the procedure were performed according to manufacturer’s guidelines. Small RNA cDNA libraries were pooled based on concentrations of each sample determined by a Qubit 4 Fluorometer using the Qubit dsDNA HS kit and based on library fragment range determined by Agilent 4150 TapeStation using Agilent High Sensitivity DNA ScreenTape kit. Sequencing was performed on Illumina NextSeq 500 using single-end high output mode with 75 cycles, 6nt index read mode with a sequencing depth of 10 million reads per sample.

### Differential expression analysis

Firstly, reads mapping to rRNA together with transcripts with less than five normalized counts in less than six samples (size of smaller group) were excluded. Final datasets consisted of 437 miR and 21,859 RNA transcript counts across the sample collection. Subsequently, DESeq2 (R package) [24] was used to apply variance stabilizing transformation (VST) on filtered counts as well as for differential expression (DE) analysis. Additionally, to enhance accuracy of our analysis and remove false positive hits, DE analysis was performed a second time using the Wilcoxon rank-sum test [25]. In both cases, the significance level of DE was defined as|log2(fold change)| ≥ 2 and FDR-adjusted *P*-value < 0.05. Differentially expressed genes (DEGs) and differentially expressed miRs (DEMs) fulfilled the following two requirements: (I) they were significantly differentially expressed in both analyses (i.e., the intersection of DESeq2 and Wilcoxon rank-sum test) and (II) the magnitude of their differential expression was the same sign in both analyses, positive or negative difference compared to the non-diseased group. Then, the final DE intersection analyses were visualized as volcano plots of the DESeq2 data. For visualization of differences in expression patterns between tumor and non-diseased samples, principal component analysis (PCA) was used. The top 10% of more and less abundant RNA transcripts and all more and less abundant miR transcripts were also hierarchically clustered and visualized as heatmaps. For hierarchical clustering, Euclidean distance and Ward's linkage criteria were used. To quantify distinction between tumor and non-diseased, clusters silhouette score was used. For PCA analysis, hierarchical clustering, and silhouette score calculation we used the scikit-learn (https://github.com/scikit-learn/scikit-learn) python library. For scatter plot and heatmap visualization, seaborn (https://github.com/mwaskom/seaborn) and matplotlib (https://github.com/matplotlib/matplotlib) python libraries were used. Gene ontology (GO) annotation and Kyoto Encyclopedia of Genes and Genomes (KEGG) enrichment analysis were performed for more/less abundant genes by The Database for Annotation, Visualization and Integrated Discovery (DAVID) annotation tool separately [26, 27, 28].

### Focused analysis of expression levels of key clinicopathological markers

In clinical practice, several biomarkers are routinely studied for accurate PanNET diagnosis and management. RNA-seq data was scrutinized for the expression of key general biomarkers (*CHGA*,* PPY*,* ENO2*,* GNRH1*), specific biomarkers important for functioning tumors (*INS*,* IGF1*,* GCG*,* VIP*,* SST*,* PTHLH*), theranostic biomarkers (*SSTR1*,* SSTR2*,* SSTR3*), immunohistochemical tissue biomarkers (*MKI67*,* CHGA*,* SYP*,* PGR*,* CCND1*,* NESP55*,* CDX2*,* PAX8*,* PDX1*) as well as known molecular drivers (*DAXX*,* ATRX*,* TP53*,* RB1*,* MEN1*,* TSC1*,* TSC2*,* MGMT*). For these biomarkers, normalized gene expression values were filtered and compared between tumor and non-diseased tissues. Mann-Whitney U test was used to assess significance.

### miR–mRNA interaction network and GO/KEGG analysis

For prediction of miR-mRNA pairs, the top three most abundant and top three least abundant miRs were selected to demonstrate the proof-of-concept of generating biologically relevant miR/mRNA interaction networks. The miR’s putative mRNA targets were predicted by the miRDB [29] in silico prediction tool with default settings. These predicted targets were then intersected with our set of DEGs, specifically comparing the targets of the most abundant miRs with the least abundant mRNA DEGs and vice versa. The purpose of selecting only the top three most and least abundant miRs was to provide a manageable yet representative sample for analyzing miR-mRNA interactions, while ensuring that the results would be meaningful and interpretable. Running a larger number of miRs through miRDB would yield a large set of targets, making it difficult to identify consistent and biologically relevant miR-mRNA pairs. These predicted targets, which were differentially expressed in PanNETs, were used for function enrichment analysis. Here, the online tool for GO [26, 27] and KEGG analysis was the module combined with clusterProfiler [30], Pathview [31], and the Scientific and Research plot tool (SRplot) [32]. These results were clarified by the DAVID annotation tool [28]. Results with *P*-value < 0.05 were considered significantly enriched. For correlation analysis of predicted targets, Pearson correlation and subsequent hierarchical clustering with Ward's linkage was used. For visualization of predicted miR-mRNA pairs as well as for degree calculation, the NetworkX (https://github.com/networkx/networkx) python library was used.

## Results

### Differential expression analysis of mRNA

**Fig. 1:**
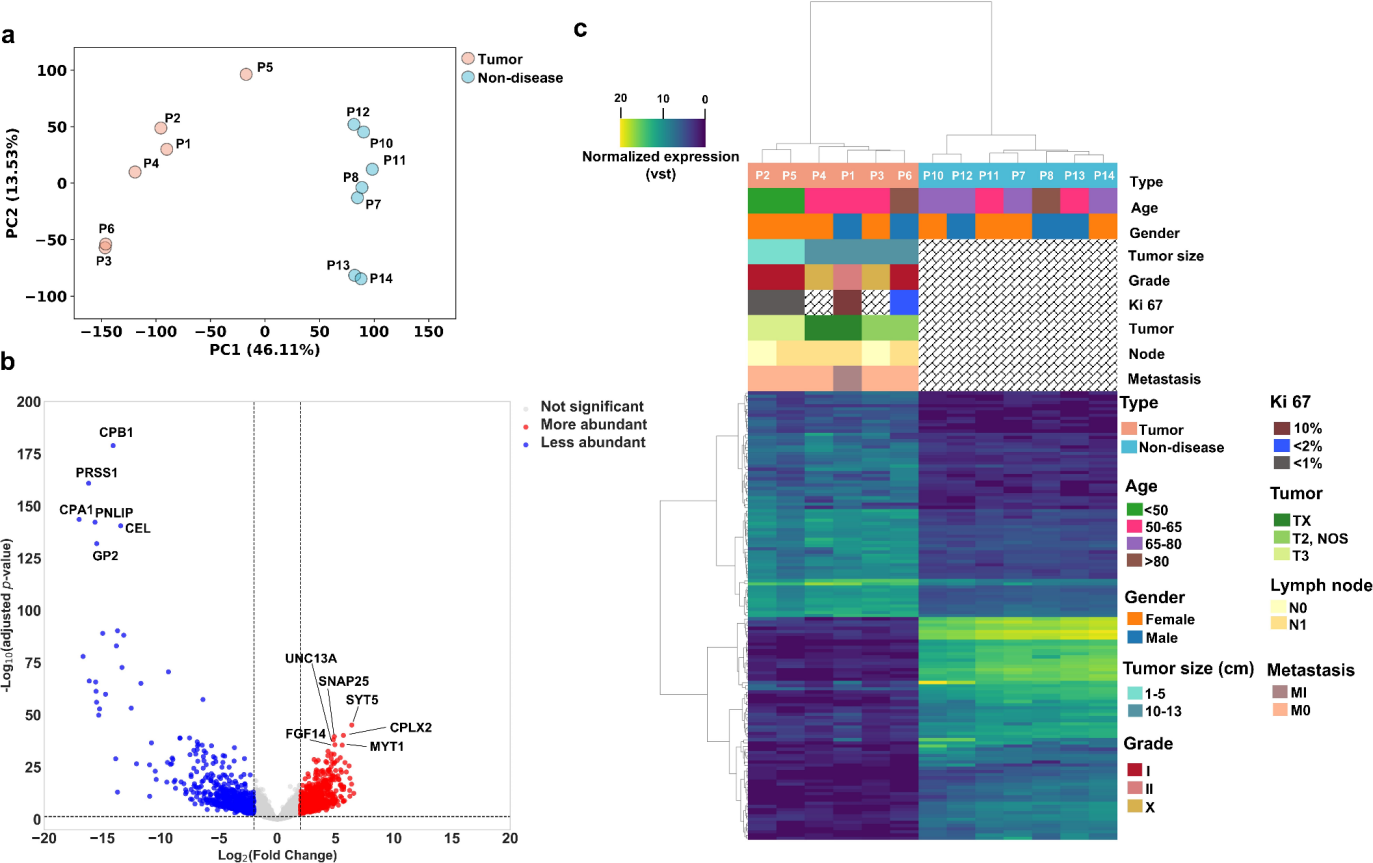
Analysis of mRNA expression levels. (**a**) PCA analysis of tumor (*n* = 6) and non-diseased (*n* = 7) tissue samples. (**b**) Volcano plot of intersection DE analysis. The significance level was set to|Log2(fold change)| ≥ 2 and - Log10(adjusted *P*-value) < 0.05. (**c**) Hierarchically clustered heatmap of top 10% more abundant and less abundant genes based on their corresponding VST values.

First, we used PCA to investigate whether transcriptomic profiles differed between the two sample groups. The plot of PC1 (46.11% variance cover) and PC2 (13.53% variance cover) showed clear differences between tumor and non-diseased samples, with no overlapping samples between groups in PC1 (Fig. 1a). Moreover, the clustering was not affected by the sex or age of patients (Supplementary Fig. S1).

To investigate differential expression patterns of mRNAs in tumor versus non-diseased groups, we took the intersection of the two DE analyses (DESeq2 and Wilcoxon rank sum test) and revealed 1,417 (7.72% of all analyzed) DEGs (Supplementary Table S2), which are divided into 715 more abundant and 702 less abundant (Fig. 1b). Hierarchical clustering of the top 10% (72) more abundant and top 10% (70) less abundant genes showed clear separation of two clusters corresponding to tumor (light orange) and non-diseased (light blue) groups (Fig. 1c). Unsupervised cluster analysis showed different clusters of tumor and non-diseased samples with a silhouette score of 0.75, indicating relatively well-separated clusters.

To shed light on the functional characteristics of the DEGs, the more abundant and less abundant DEGs were analyzed using GO and KEGG enrichment analyses separately. (Supplementary Table S3 and Supplementary Table S4). A list of the top 10 GO terms from the three categories biological process (BP), cellular component (CC), and molecular function (MF) is presented in Supplementary Fig. S2. For the PanNET less abundant DEGs, the GO analysis showed that DEGs were significantly enriched in digestion at the BP level, in extracellular exosome at the CC level and in serine– type endopeptidase activity at the MF level. Furthermore, the enriched KEGG pathways of the less abundant DEGs included pathways in pancreatic secretion, protein digestion and absorption, maturity onset diabetes of the young, metabolism of xenobiotics by cytochrome P450 and metabolic pathways. For the PanNET more abundant DEGs, the GO analysis showed that DEGs were significantly enriched in regulation of ion transmembrane transport at the BP level, in dendrite at the CC level and in calcium ion binding at the MF level. Furthermore, the enriched KEGG pathways of the more abundant DEGs included pathways in dopaminergic synapse, GABAergic synapse, circadian entrainment, glutamatergic synapse, and insulin secretion.

### Biomarker expression and association with key clinicopathological markers

**Fig. 2:**
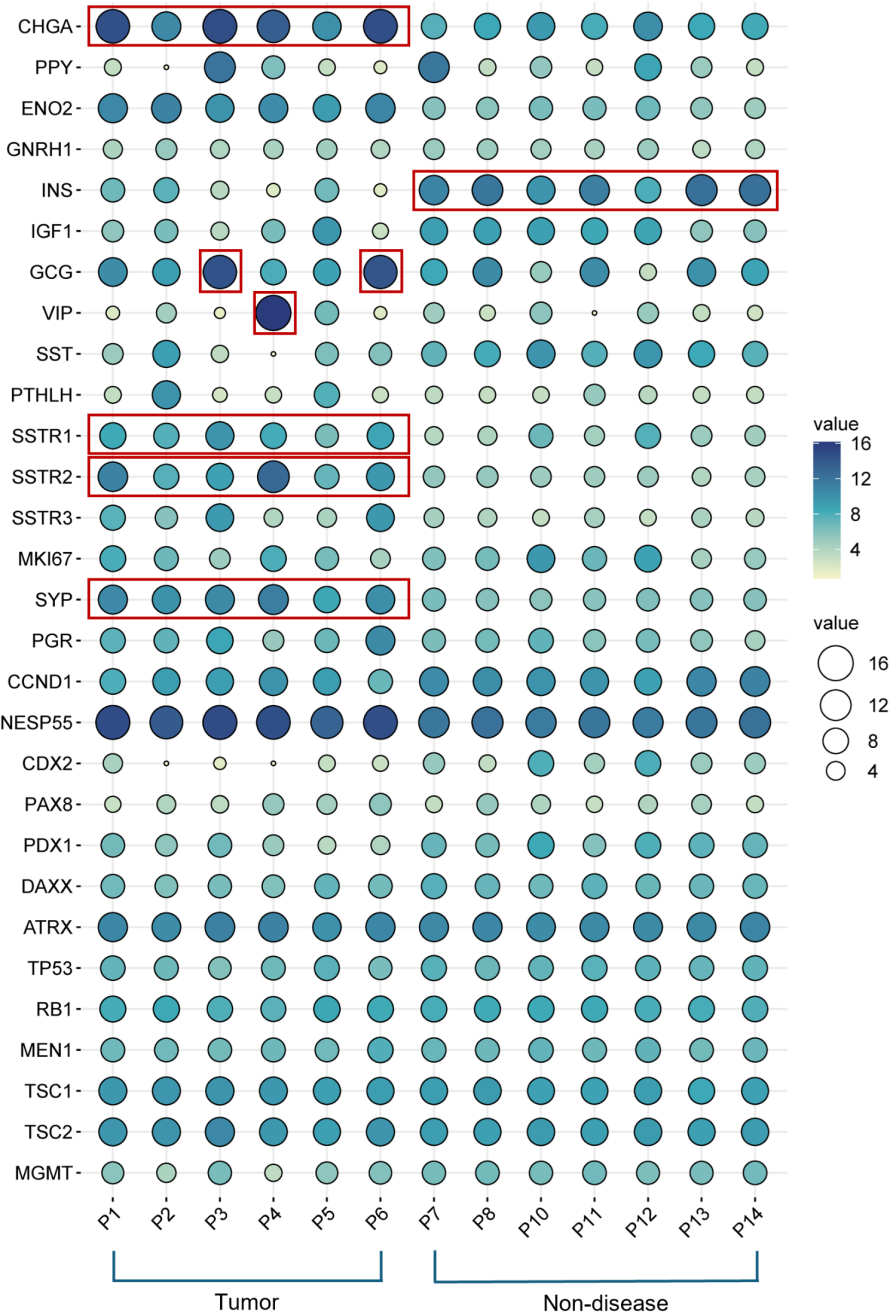
Clinically relevant mRNA biomarker expression. The balloon plot shows the expression of key biomarkers in individual PanNETs compared to non-diseased tissue. Both the heatmap and size of dots denote normalized gene expression (VST). Red rectangular boxes highlight key markers assessed in routine clinical practice.

We used RNA-seq to assess the expression of clinically relevant biomarkers for PanNET management in tumor compared to non-diseased tissues (Fig. 2) and then associated these findings with known clinicopathological data.

Key biomarkers, including chromogranin A (*CHGA*,* P* < 0.001) and synaptophysin (*SYP*,* P* < 0.001), were differentially expressed in tumors compared to non-diseased control tissues. This suggests that RNA could serve as a surrogate analyte for these markers commonly used for PanNET diagnosis via IHC. Similarly, mRNA for the somatostatin receptors, *SSTR1* (*P* < 0.01) and *SSTR2* (*P* < 0.001), which are associated with somatostatin analogue therapy, were differentially expressed across all tumor samples. In addition, sample P4, identified as a tumor secreting vasoactive intestinal peptide (VIP) based on biobank data, showed a substantial increase in *VIP* mRNA expression. Interestingly, non-diseased samples exhibited higher levels of insulin (*INS*, *P* < 0.001) expression compared to tumors. Increased abundance of glucagon (*GCG*) was observed in tumor samples P3 and P6. Moreover, most of the samples in our cohort display a low IHC Ki-67 index (P1: 10%, P2: <1%, P5: <1%, P6: <2%), which is consistent with our *MKI67* mRNA expression data. These findings demonstrate that mRNA expression data can offer a streamlined way to profile many clinically relevant biomarkers using a single approach, yielding valuable insights into the molecular profile of PanNETs that complements traditional diagnostic and prognostic biomarkers.

### Differential expression analysis of miRs

**Fig. 3:**
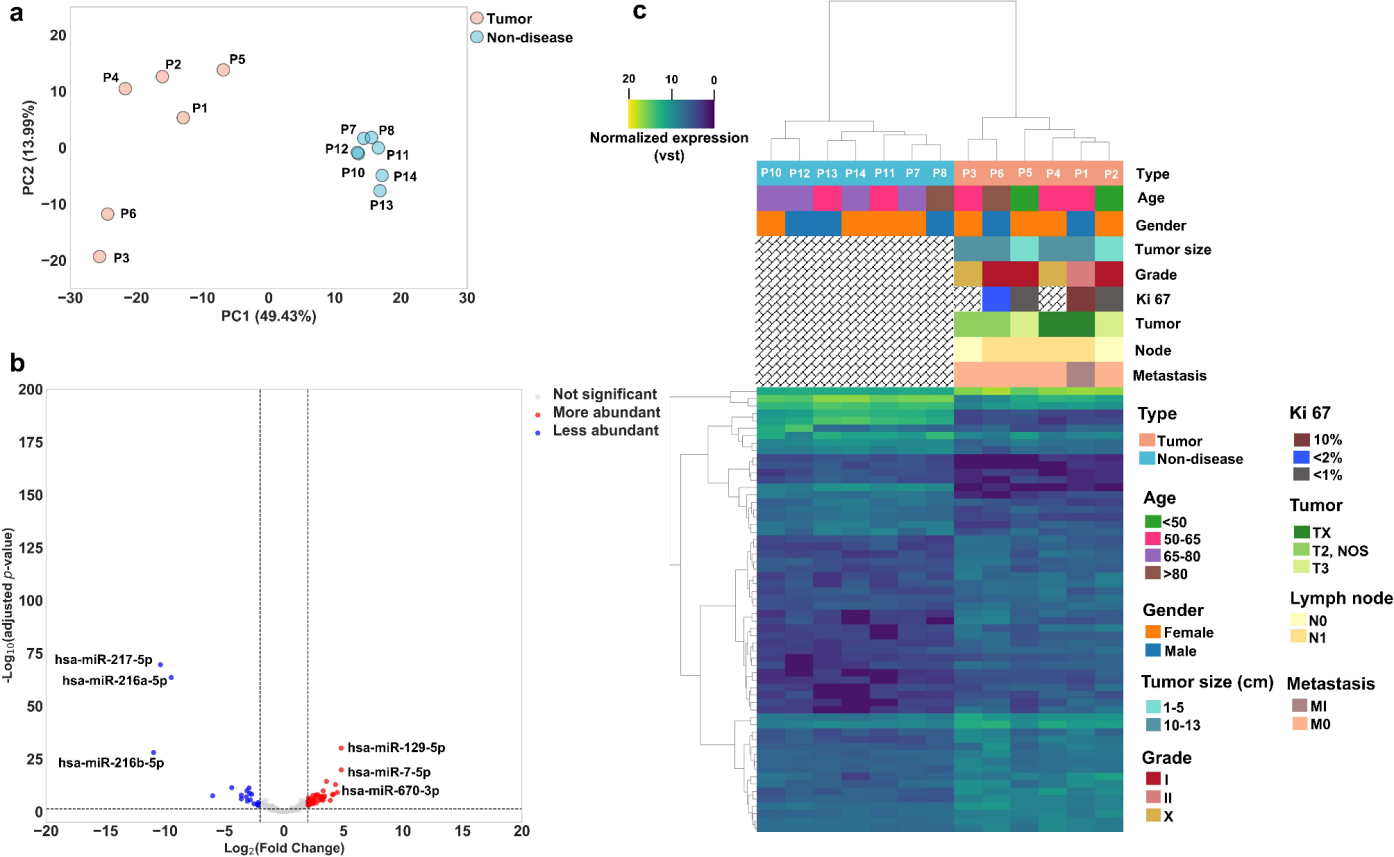
Analysis of miR expression levels. (**a**) PCA analysis of tumor (*n* = 6) and non-diseased (*n* = 7) tissue samples. (**b**) Volcano plot of intersection DE analysis. The significance level was set to|Log_2_(fold change)| ≥ 2 and - Log10(adjusted *P*-value < 0.05. (**c**) Hierarchically clustered heatmap of all (60) DEMs based on their corresponding VST normalized values.

Similar to mRNA, PCA analysis of miRNome profiles (Fig. 3a) showed clear differences in expression patterns between tumor and non-diseased groups in the first two dimensions, PC1 (49.43% variance covered) and PC2 (13.99% variance covered), which were independent of the patients’ sex or age (Supplementary Fig. S3).

To investigate differential expression patterns of miRs in tumor versus non-diseased groups, we again used the intersection of two DE analyses and found 60 (16.6%) miRs with 41 and 19 miRs more abundant and less abundant, respectively (Fig. 3b). Hierarchical clustering of all DEMs yielded two different clusters for tumor and non-diseased samples, although the separation (0.57 silhouette score) was less defined than the clusters based on mRNA profiles.

The miR-216 cluster (miR-216a, miR-216b, and miR-217), located in the same genomic region of chromosome 2 (2q16.1), were the least abundant miR in tumor versus non-diseased groups; Conversely, miR-670-3p, miR-129-5p, and miR-7-5p were the most abundant miRs in tumor versus non-diseased groups (Supplementary Table S5).

### Construction of the miR-mRNA interaction network

**Fig. 4:**
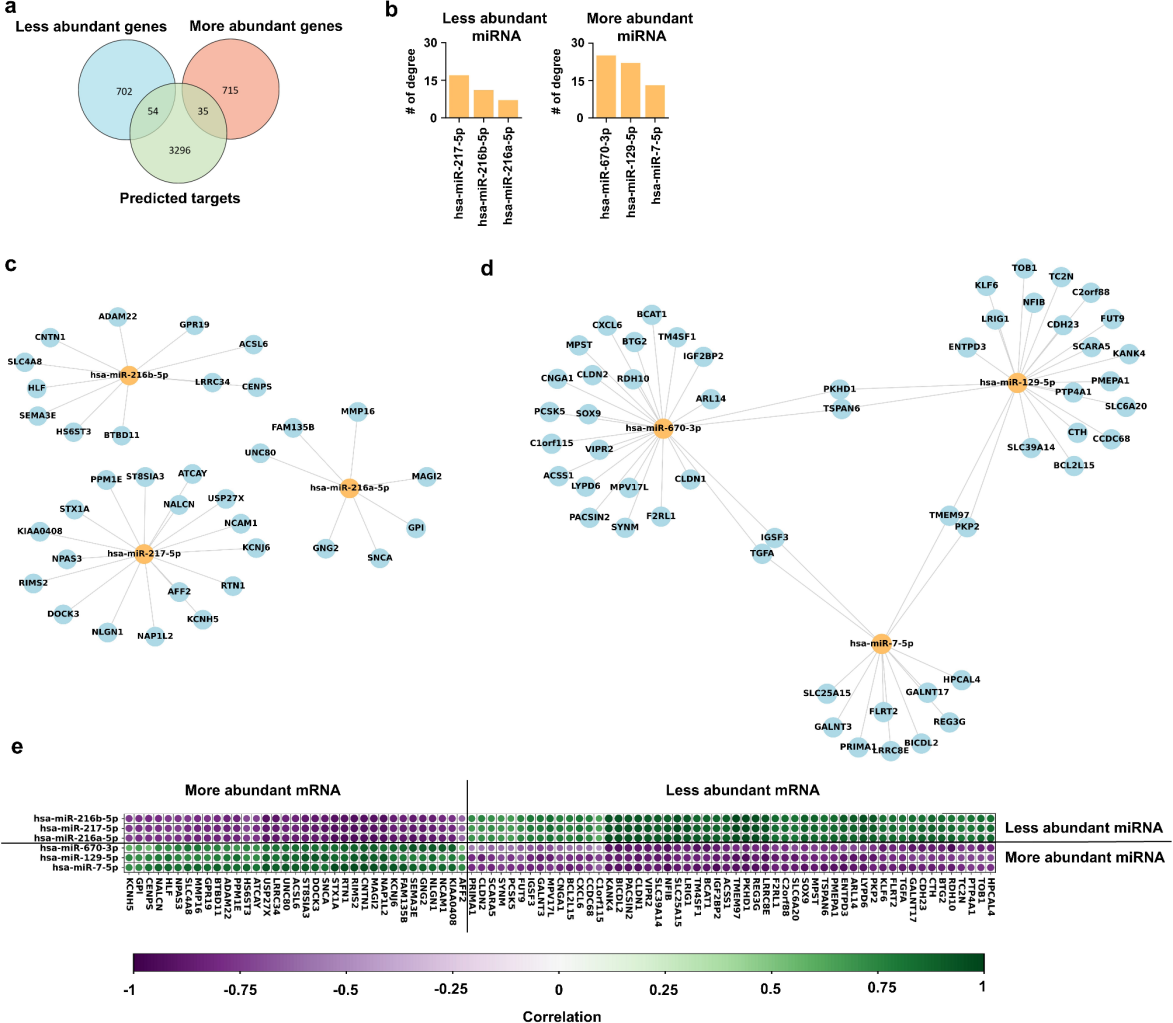
Putative miR-mRNA interaction networks. (**a**) Venn diagram of the less abundant and more abundant DEGs and predicted targets. (**b**) Histograms of degree counts for top three least abundant DEMs (left) and top three most abundant DEMs (right). (**c**) Network of top three least abundant DEMs with predicted more abundant mRNA. (**d**) Network of top three most abundant DEMs with predicted less abundant mRNA targets. (**e**) Hierarchically clustered correlation dot–plot between selected miR and all their corresponding predicted mRNA pairs.

Given that PanNETs can be stratified into molecular subtypes by their separate mRNA and miR profiles [23], we aimed to extend these findings by identifying miR-mRNA interaction networks using the top three most abundant miRs: 670-3p, 129-5p, and 7-5p as well as the top three least abundant miRs: 216a-5p, 216b-5p, and 217-5p. Then, we used the miRDB [29] target prediction tool with default settings to generate a list of putative mRNA targets (Supplementary Table S6) and overlapped the list of putative mRNA targets with our list of DEGs (Fig. 4a). As a result, we found 54 unique targets for more abundant miRs and 35 unique targets for less abundant miRs. More abundant miR-670-3p had the most targets (*N* = 25), while the less abundant miR-216a-5p had the least targets (*N* = 7) in PanNETs versus non-diseased tissues (Fig. 4b). The network of less abundant miRs (Fig. 4c) consists of three distinct sub-networks with no shared targets among them, while our more abundant miRs (Fig. 4d) shared six mRNA targets together, specifically *PKHD1* and *TSPAN6* between miR-670-3p and miR-129-5p. Also, miR-129-5p share *TMEM97* and *PKP2* with miR-7-5p, and miR-7-5p share targets *IGSF3* and *TGFA* with miR-670-3p.

For a more in-depth analysis of the relationships between expression patterns of miR-mRNA pairs, we calculated Pearson correlation coefficients between the selected DEMs, and all their predicted mRNA targets (*N* = 89), which participate in 95 interactions (Fig. 4e). Correlation between more abundant miRs and their less abundant mRNAs are generally strongly negative with the range of R (-0.95, -0.47), with the highest anticorrelation for miR-670-3p/*RDH10* and miR-129-5p/*PKHD1* pairs and the smallest anticorrelation between the pairs miR-670-3p/*C1ORF115* and miR-670-3p/*CLDN2* (Supplementary Table S6). Counterintuitively, all more and less abundant miRs have some putative mRNA targets that are expressed in the same direction (i.e., both the increased abundance of the miR and its putative target), suggesting that regulation at the transcript level may occur through an indirect mechanism or direct regulation may not result in degradation of the mRNA transcript, but rather translational repression.

### GO term & KEGG pathway enrichment analysis of target genes

**Fig. 5:**
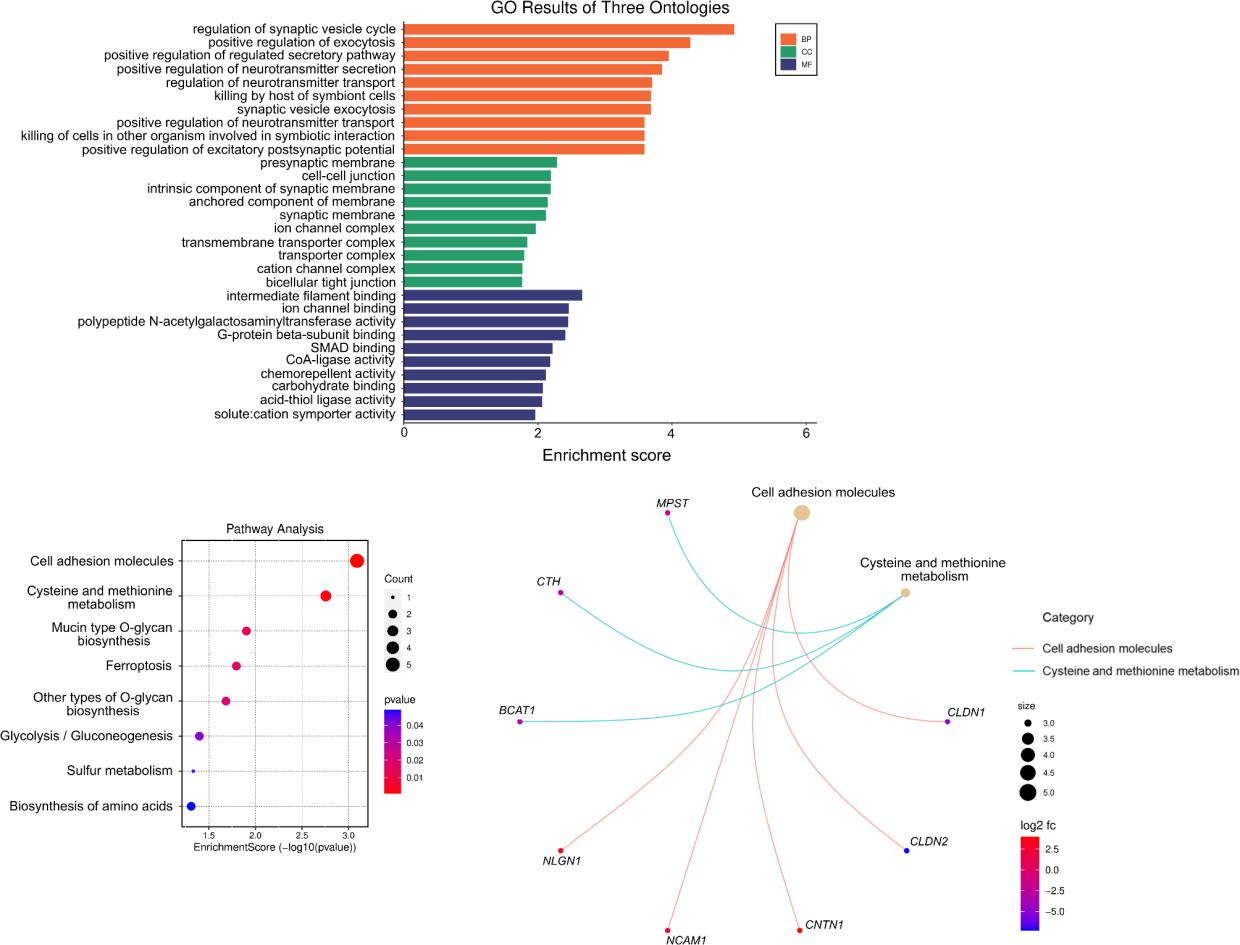
Function analysis of miR-mRNA interaction network. (**a**) GO enrichment analysis and (**b**) KEGG pathway analysis of the 89 predicted gene targets (dot plot on the left, network plot on the right). The size and color of the dots represent the amount of DEGs enriched in the pathway and enrichment significance.

To elucidate the potential function of candidate target genes (89) observed in our miR-mRNA interaction networks, we performed GO and KEGG pathway enrichment analyses (Supplementary Table S7). In biological processes, the enriched GO terms were associated with regulation of synaptic vesicle cycle, positive regulation of exocytosis and positive regulation of regulated secretory pathway (Fig. 5a). These targeted genes were categorized into the Cell adhesion molecules pathway (*NLGN1*,* NCAM1*,* CNTN1*,* CLDN1* and *CLDN2*), Cysteine and methionine metabolism (*MPST*,* CTH* and *BCAT1*), Mucin and other type O-glycan biosynthesis (*GALNT17* and *GALNT3*), Ferroptosis (*SLC39A14* and *ACSL6*), Glycolysis/Gluconeogenesis (*ACSS1*/*GPI*) and Sulfur metabolism (*MPST*) by the KEGG pathway analysis (Fig. 5b).

## Discussion

Current diagnostic biomarkers for PanNETs are limited to individual immunohistochemical markers and a handful of mutated genes, making it challenging to capture the complex and heterogeneous nature of PanNETs. In this study, we sequenced both mRNA and miR, integrated these results with in silico miR target predictions, and identified novel dysregulated mRNA-miR interaction networks in PanNETs compared to non-diseased pancreatic samples. Our comprehensive analysis of PanNET coding and small non-coding transcriptomes provides valuable biological insights into regulatory mechanisms of PanNETs, thus demonstrating the feasibility of combining mRNA/miR profiling. This provides a foundation for future studies on larger cohorts that could explore PanNET pathogenesis with the aim of revealing novel biomarkers and potential therapeutic targets.

While emerging evidence underlines the importance of both mRNA and miR transcriptomes in PanNETs, we found only one study describing pairwise next-generation sequencing analysis of mRNA/miRs and their interaction networks in PanNETs [33]. Our approach extends these findings by (1) using a well described, homogeneous clinical cohort of low-mid grade (G1-2), high risk (large tumor, node positive), well differentiated PanNETs; (2) conducting stringent differential expression analysis using two combined approaches to mitigate false positive DEGs; and (3) performing transcriptome profiling of mRNA/miRs on identical RNA samples from the same patients, enabling robust investigation of miR-target interactions.

To construct a miR-mRNA regulatory network, we focused on the top three least abundant (miR-216a-5p, miR-216b-5p, and miR-217-5p) and the three most abundant (miR-670-3p, miR-129-5p, and miR-7-5p) miRs and generated high probability mRNA targets by crossing lists of miR in silico putative mRNA targets with PanNET-specific DEGs. Although several studies have reported downregulation of the miR-216 family [33] and upregulation of miR-7-5p and miR-129-5p in neuroendocrine tumors (NETs) [34, 35, 36, 37], including PanNETs, we extend these findings by highlighting the interconnections with mRNA targets, biological processes, and pathways. Our functional analyses of miR-mRNA networks yielded enriched GO terms that were consistent with aberrations in neuroendocrine cells and overactive secretion of bioactive hormones.

The KEGG pathway analysis revealed enrichment in the cell adhesion molecule (CAM) pathway, which included promising miR-mRNA pairs such as miR-217-5p/*NLGN1*, miR-217-5p/*NCAM1*, and miR-216b-5p/*CNTN1*. In NETs, CAMs are involved in tumor progression, metastasis, and interactions with the tumor microenvironment [38, 39, 40]. The *NCAM1* (Neural Cell Adhesion Molecule 1) and *CNTN1* (Contactin 1) genes are members of the immunoglobulin superfamily (IgSF) of cell adhesion molecules involved in cell-cell interactions, neuronal development, and synaptic plasticity [41]. *NCAM1*, also known as *CD56*, has been used as an IHC diagnostic and prognostic biomarker with high sensitivity and specificity to differentiate PanNETs from other pancreatic cancers [42, 43, 44, 45]. Because *NCAM1* plays an important role in cell adhesion, detachment, and cell aggregation, tumors that cannot express *NCAM1* tend to grow rapidly, and adjacent invasion is more common [44]. Negative regulators of *NCAM1*, such as miR-217-5p, may serve as important therapeutic targets to ensure high *NCAM1* expression and prevent the invasive potential of PanNETs.

We observed a novel miR-mRNA interaction between miR-670-3 and two claudins (*CLDNs*), *CLDN1* and *CLDN2*, that were part of an enriched cell adhesion molecule KEGG pathway. Dysregulated expression of *CLDNs* is common in many cancers and plays a role in tumor initiation, progression, metastasis, and treatment response. While PanNETs show high expression of *CLDNs − 3* and − *7* and low expression of *CLDNs − 1* and *− 4*, pancreatic ductal adenocarcinomas express *CLDNs − 1*,* -4*, and *− 7* [46], indicating that unique claudin signatures may serve as therapeutic targets and biomarkers to distinguish pancreatic cancers of different cellular origins (i.e., endocrine vs. exocrine). Our results showed that PanNETs have less abundant *CLDN1* and *CLDN2*, two putative target genes of the more abundant miR-670-3p. In addition, we observed a decrease in the abundance of *CLDN10*, *CLDN18*, and *CLDN23* in our DEGs (Supplementary Table S2). Since the increased abundance of miR-670-3p in PanNETs is novel and yet to be reported in the literature, this warrants additional investigation to confirm that claudins are a direct target of mir-670-3p as well as to elucidate the precise roles of individual claudin isoforms in PanNET pathogenesis.

Our mRNA-miR interaction networks discovered novel pairs (miR-670-3/*BCAT1*, miR-670-3/*MPST*, miR-129-5p/*CTH*) that play a key role in the cysteine and methionine metabolism pathway, suggesting alterations in metabolic processes in PanNETs. A recent multiomic (transcriptomic and metabolomic) analysis of duodenopancreatic NETs showed that BCAT1 expression was associated with intermediate outcomes and *CTH* (cystathionine gamma-lyase) gene enrichment in glutathione synthesis in PanNETs [47]. The *CTH* gene has been studied in various tumors, including lung adenocarcinoma [48], prostate cancer [49], hepatocellular carcinoma [50], and glioblastoma [51]; however, its specific expression in PanNETs remains unknown. While the role of *MPST* (also known as *3-MST*) in PanNETs is unclear, there is growing evidence that hydrogen sulfide producing enzymes like MPST are more abundant in numerous cancers and may serve as druggable targets [52, 53]. Overall, genes involved in the cysteine and methionine metabolic pathway play critical roles in cancer pathogenesis by influencing regulation of redox balance, antioxidant defense, and cellular proliferation in cancer cells [54]. Our results support this emerging evidence of distinctive metabolic gene programs in PanNETs and suggest that perturbed expression of miR-mRNA networks may be implicated in altered regulation of genes and pathways involved in energy homeostasis.

Consistent with our results, multiple studies report more abundant miR-7-5p in NETs [34, 35, 36, 37]. In comparison with non-NET controls, miR-7 levels are 48-fold higher in all NET cases [35], suggesting that miR-7 has a high degree of neuroendocrine specificity. Another study in gastroenteropancreatic neuroendocrine tumors indicates that upregulation of miR-7-5p can inhibit cell proliferation and induce apoptosis [55]. One potential mechanism through which miR-7 may influence pancreatic neuroendocrine progression, is by regulating expression of regenerating islet-derived (*Reg*) gene family members expressed in pancreatic endocrine cells. For example, miR-7 directly targets mouse *Reg1* expression in pancreatic acinar and islet β-cells [36]. Interestingly, our data shows a significant reduction of Reg family members (*REG1A/B*, *REG3A/G*, and *REG4*), which could be driven by the increased abundance of miR-7-5p. *REG3G* was also found in the interaction hub of DEGs that were predicted targets of miR-7-5b. Additionally, *REG1A* and *CPA1* show utility in diagnosing mixed pancreatic acinar cell carcinoma/acinar-neuroendocrine carcinoma [56]; notably, we show that *CPA1* has the highest reduction in PanNETs, which is consistent with the absence of *CPA1* immunohistostaining in PanNETs [57]. We also observed miR-7-5p as a key player in an mRNA-miR interaction network that included coregulation of *TMEM97* and *PKP2* with miR-129-5p and *IGSF3* and *TGFA* with miR-670-3p. Future studies should aim to elucidate the mechanisms behind the increased abundance of miR-7-5p expression and how this may regulate cell-type specific pancreatic gene networks (e.g., *Reg* family) and how this could be exploited for therapeutic and diagnostic purposes.

This study also examined mRNA expression of biomarkers in PanNETs and compared it with clinical data to better understand the disease’s molecular characteristics and prognosis. Elevated levels of *CHGA* and *SYP* in PanNETs indicate increased neuroendocrine cell production, which could help identify tumors and predict their aggressiveness. Increased *SSTR2* expression suggests potential sensitivity to somatostatin, with therapeutic implications for somatostatin analogues in PanNET management. Additionally, mRNA analysis confirmed the VIP-secreting tumor diagnosis for patient P4 and suggested that patients P3 and P6 may have a rare form of PanNET, glucagonoma. This suggests that whole transcriptome sequencing could be used to profile clinically relevant PanNET biomarkers. As sequencing becomes more cost-effective and efficient, this approach could become a viable option for diagnostic applications in clinical settings.

The current study is inherently affected by several limitations. First, the study’s sample size is relatively small, making it important to confirm the validity of these findings in larger cohorts. Second, although clinical data were integrated into the analysis, select information was missing, including certain IHC biomarkers due to them not being part of routine diagnostics and long-term outcome data. Thus, the study focused primarily on molecular profiling. Further validation of the clinical significance of the identified biomarkers and pathways is required in independent cohorts with detailed longitudinal outcome data. Third, the non-diseased pancreatic control tissue likely contains an exocrine component, thus the differentially expressed genes may reflect both the endocrine and exocrine composition of the control tissue.

Our study provides a proof-of-concept demonstration that mRNA and miR sequencing can be used to interrogate clinically relevant biomarkers for PanNETs. The identification of novel miR-mRNA pairs and interaction networks that are central to altered neuroendocrine function, cell adhesion, and metabolic pathways underscores how this approach can yield insights into biological processes and signaling pathways important for PanNET biology. Further functional characterization of these miR-mRNA networks as well as individual DEGs in larger cohorts may reveal new biomarkers and therapeutic targets for the diagnosis and treatment of PanNETs, ultimately paving the way for future research aimed at improving the outcomes of patients with PanNETs.

## Electronic supplementary material

Below is the link to the electronic supplementary material.

Supplementary Material 1

Supplementary Material 2

## Data Availability

The mRNA-seq and miRNA-seq datasets generated during the current study are available in the Gene Expression Omnibus (GEO) repository, under accession numbers GSE280271 (mRNA-seq), https://www.ncbi.nlm.nih.gov/geo/query/acc.cgi?acc=GSE280271 and GSE279952 (miRNA-seq), https://www.ncbi.nlm.nih.gov/geo/query/acc.cgi?acc=GSE279952. Additional data is provided within the manuscript or supplementary information files.

## List of Abbreviations

3’UTR: 3’untranslated region
BP: biological process
CAM: cell adhesion molecule
CC: cellular component
CLDNs: claudins
DAVID: The Database for Annotation, Visualization and Integrated Discovery
DE analysis: for differential expression analysis
DEGs: differentially expressed genes
DEMs: differentially expressed miRs
FDR: false discovery rate
FFPE: Formalin-Fixed Paraffin-Embedded
GO: Gene ontology
IgSF: the immunoglobulin superfamily
IHC: immunohistochemistry
KEGG: Kyoto Encyclopedia of Genes and Genomes
MF: molecular function
miRs: microRNA
mRNAs: messenger RNAs
NETs: neuroendocrine tumors
PanNETs: pancreatic neuroendocrine tumors
PCA: principal component analysis
UD index: Unique dual index
VIP: vasoactive intestinal peptide
VST: variance stabilizing transformation
