# Supplementary material for: Deep sequencing reveals distinct microRNA-mRNA signatures that differentiate pancreatic neuroendocrine tumor from non-diseased pancreas tissue
Source: BMC Cancer. 2025 Apr 11;25:669. doi: 10.1186/s12885-025-14043-w (PMC11987397; doi:10.1186/s12885-025-14043-w)
Supplement: Supplementary file 1 — Supplementary Material 1 [file 12885_2025_14043_MOESM1_ESM.pdf]

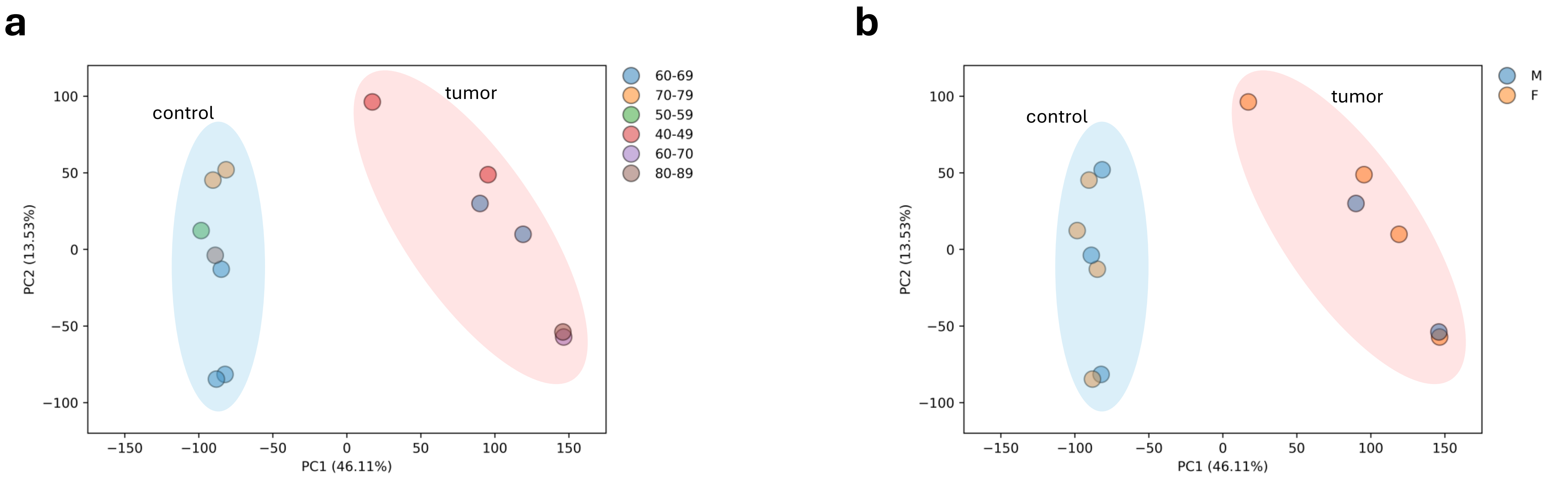

Supplementary Figure S1. PCA plot of RNA transcripts colored by (**a**) age and (**b**) sex in tumor and non-diseased samples.

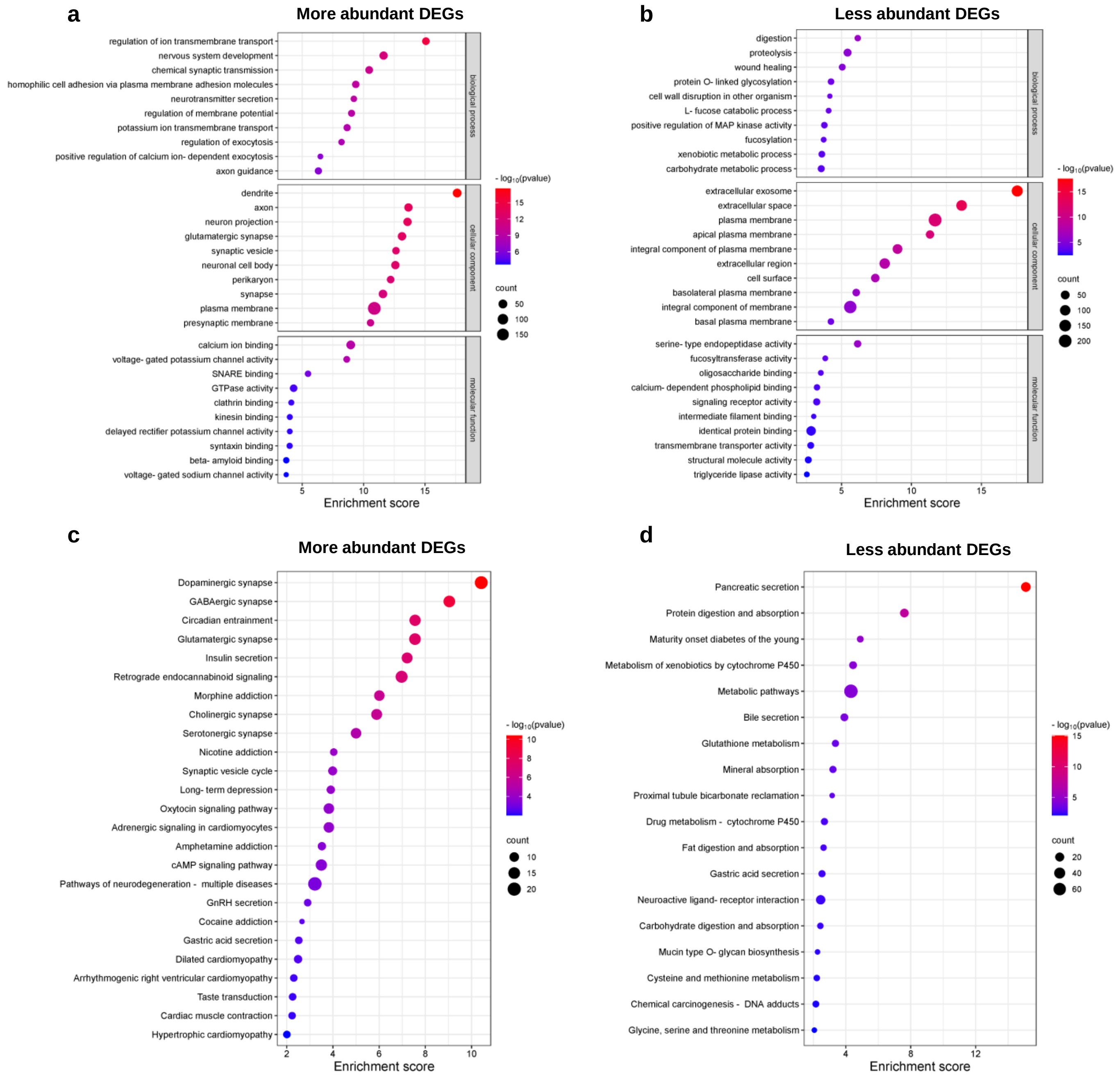

Supplementary Figure S2. GO and KEGG functional enrichment analysis of 1,417 differentially expressed mRNAs. (**a**) GO analysis of more abundant DEGs (**b**) GO analysis of less abundant DEGs. (**c**) Pathway enrichment analysis of more abundant DEGs (**d**) Pathway enrichment analysis of less abundant DEGs (GO, Gene Ontology; DEGs, differentially-expressed genes; KEGG, Kyoto Encyclopedia of Genes and Genomes)

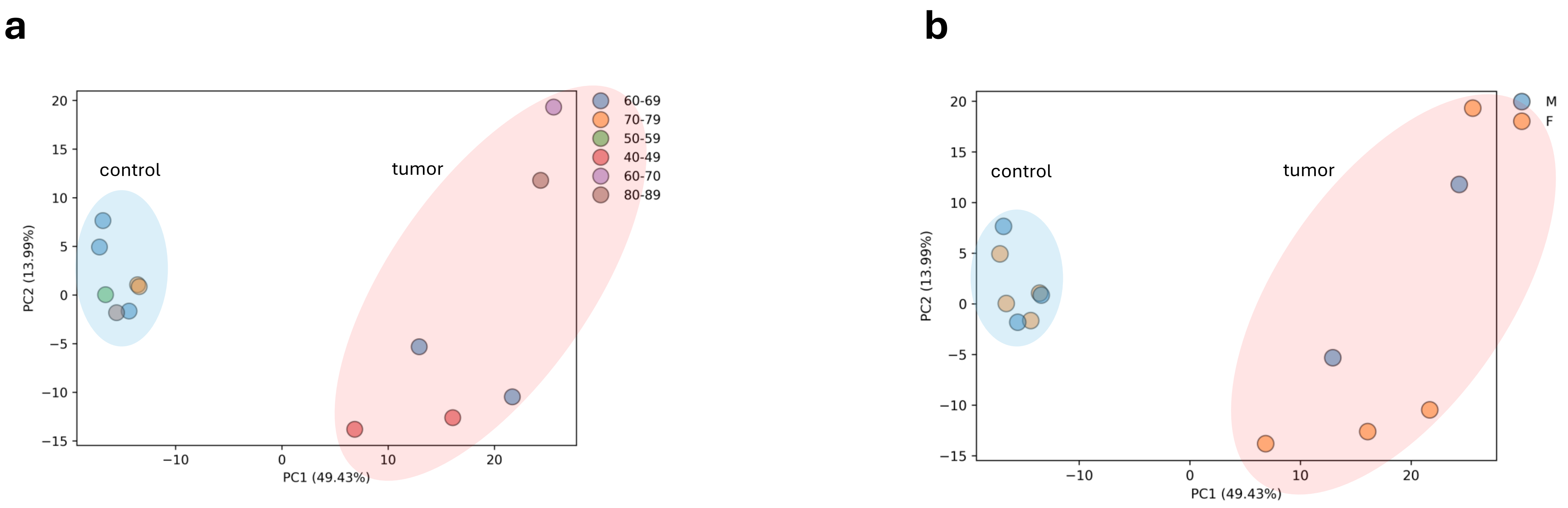

Supplementary Figure S3. PCA plot of miR transcripts colored by (**a**) age and (**b**) sex in tumor and non-diseased samples.
